# Integrated Dementia Care in the DementiaNet Program: Health Economic Reflections on Interpretation, Assessment, and Evaluation: Comment on "Effects of DementiaNet’s Community Care Network Approach on Admission Rates and Healthcare Costs: A Longitudinal Cohort Analysis"

**DOI:** 10.34172/ijhpm.9301

**Published:** 2025-09-08

**Authors:** Ansgar Wübker

**Affiliations:** ^1^Hochschule Harz, Wernigerode, Germany.; ^2^RWI – Leibniz Institute for Economic Research, Essen, Germany.

**Keywords:** Integrated Dementia Care, Health Economics, DementiaNet, Cost Shifting, Observational Methods

## Abstract

This commentary discusses the study by Remers et al. The authors analysed the impact of the Dutch DementiaNet programme on hospital admissions and healthcare costs for individuals with dementia. Using detailed claims data of over 38 000 insured individuals, the study found that participation in DementiaNet networks was associated with fewer hospital admissions and care days, as well as reduced hospital costs. Furthermore, participation in DementiaNet was linked to increased outpatient healthcare expenditure, while overall healthcare costs remained stable. This commentary seeks to place the findings within health economic theory. It posits that DementiaNet could reduce information asymmetries, transaction costs and disincentives in dementia care. Through its network- and primary care-based approach, DementiaNet plausibly improves care coordination, which might enable earlier interventions. This could account for the shift in costs from inpatient to outpatient care. Additionally, the commentary addresses methodological considerations, limitations, and directions for future research.

## Summary and Classification of the Paper

 The study by Remers et al^1^ addresses an important and previously under-researched empirical question: What impact does implementing regional, primary care-based networks for people with dementia have on hospital admissions and healthcare expenditure? Using comprehensive data on health and pension insurance claims, the authors compare individuals enrolled in local “DementiaNet” networks with a control group, based on a retrospective longitudinal cohort of more than 38 000 insured individuals in the Netherlands (2015–2019). DementiaNet is a Dutch primary care–based program which establishes local interprofessional networks in order to improve coordinated care for people with dementia. Background information on the “DementiaNet” programme can be found in Nieuwboer et al^2^ and Richters et al.^3^ The key result is as follows: Participation in the DementiaNet program is associated with a reduced risk of hospital admissions, especially to intensive care unit admission. Furthermore, participation is linked to significantly fewer inpatient care days in hospitals. While total healthcare costs did not significantly decrease, the share of hospital costs dropped notably, accompanied by a slight increase in primary care expenditures.

 The study makes an innovative contribution to the literature by investigating the real-world effects of a cross-sectoral, network-based care model for dementia using detailed longitudinal data from Dutch health and long-term care insurers. The focus is on core health economic outcomes such as hospital admission rates and costs. Methodologically, the paper is convincing through its four-year longitudinal analysis, supported by sophisticated regression models with extensive control for relevant covariates. Additional sensitivity analyses assess the robustness of the findings. Compared to the international literature,^4,5^ the study shows that not only improved interprofessional coordination, but particularly an integrated care model embedded in primary care, can generate cross-sectoral effects. It thus complements existing evidence, which has mainly focused on specialized clinical settings or traditional case management approaches. In contrast, Remers et al^1^ offer a practice-oriented perspective on primary care–based network structures.

 Given the strong increase of dementia-related care needs and the enormous pressure on financial and workforce resources, the relevance of this research is extremely high.^6^ The examined model appears particularly transferable to healthcare systems with a strong primary care foundation, such as the Netherlands or the United Kingdom. It may also be scalable to countries like Germany, where the role of the general practitioner as a coordinating entity (“Gatekeeper”) is becoming increasingly important.^6^ In such contexts, network-based, primary care–oriented structures like DementiaNet might be implemented relatively well and integrated into existing care pathways.

## Theoretical Embedding and Mechanisms of Integrated Care Networks

 Remers et al^1^ empirically demonstrate that participation in the Dutch DementiaNet programme, which fosters local interprofessional care networks for people with dementia, is associated with fewer institutional admissions, particularly to hospitals and emergency care units. It is also associated with reduced hospital-related costs. These empirical findings might be interpreted with the help of key health economic mechanisms. Thus, in the following I will present arguments from four complementary theoretical models on how integrated care networks might impact healthcare utilization and costs.

 First, as Arrow^7^ pointed out in his seminal contribution, healthcare markets are characterised by pronounced information asymmetries. Health professionals such as general practitioners or nurses often rely on incomplete information about the health status of their shared patients. DementiaNet specifically deals with this structural opacity. Regular interprofessional case discussions, topic-specific training and coordinated collaboration within local care networks create institutionalised communication spaces in which relevant information is systematically shared. In this way, the existing information asymmetry between those involved is significantly reduced. Improved coordination, particularly in dementia care, enables early and targeted interventions in the event of emerging health crises, functional decline or psychosocial stress, which may help explain the observed reductions in hospital and emergency care admissions. At the same time, it helps prevent information loss and reduce the duplication of services and inefficient parallel care.

 Second, the positive effects of the network can also be explained using the theory of transaction cost economics.^8^ Dementia care involves many different people, resulting in high coordination costs. Without structured communication processes in place, frictional losses may occur, for example due to inefficient medication coordination or conflicting treatment plans. DementiaNet may reduce these costs by providing standardised communication channels and allocating time and resources more efficiently through clearly defined responsibilities. However, it should be noted that using such networks takes time away from direct nursing and medical care.

 Third, principal–agent theory^9^ stresses the enormous importance of eliminating information asymmetries and negative incentives between principals (eg, cost bearers)and agents (eg, service providers). When care structures are highly fragmented, providers lack incentives to coordinate care effectively across sectors. The problem is that they rarely benefit financially from the efficiency gains or cost savings that integrated care can offer. This can lead to suboptimal or uncoordinated service provision. Programs such as DementiaNet may reduce information asymmetries and thus improve service delivery.

 Although DementiaNet does not impose formal governance structures, it raises a form of ‘soft’ or intermediate governance: local networks act as collective agents with jointly defined care goals and shared responsibility. Through peer monitoring, the use of quality indicators and continuous reflection within the network, decisions are increasingly oriented towards overall care outcomes, such as preventing health shocks and their associated high costs. This curbs opportunistic behaviour and mitigates systemic incentive distortions.

 Fourth, contract theory^10^ also provides an explanation of the advantages of networks such as DementiaNet. Highly complex, person-dependent services such as dementia care cannot be fully regulated by formal contracts. Therefore, DementiaNet does not rely on formal contracting. Instead, trust, reputation, implicit norms, and continuous exchange form the basis of cooperative behaviour. For interprofessional, patient-centred tasks, this form of governance may be more effective than traditional care models as it allows for flexibility and reliability.

 It should be noted that these four theoretical models or perspectives and the arguments that can be derived from them are not mutually exclusive. Rather, they complement each other. For instance, the reduction in institutional admissions observed may suggest an improvement in the early detection of health risks and more proactive care management, which could be facilitated by regular interprofessional network meetings and shared decision-making processes. Furthermore, establishing the networks may have altered the decision-making architecture. Interprofessional collaboration may have helped to ensure more consistent adherence to medical standards, reduce isolated case-by-case decisions and decrease variability in care delivery. This has likely contributed to the professionalisation of care processes and a qualitative improvement in care, particularly in critical situations.

 The observed increase in primary care costs can also be interpreted in light of theoretical considerations. Higher outpatient spending may reflect an intended substitution effect, whereby costly inpatient treatment is replaced by preventive, early, low-threshold and patient-centred interventions. While this may represent an efficient reallocation of resources towards coordinated care, it could also indicate a cost shift without clear benefits to society, especially given the difficulty of measuring certain costs in the outpatient sector, such as informal caregiving, which is arguably more relevant here than in inpatient care.

 Although from a health economic perspective cross-sectoral care models must be accompanied by similarly cross-sectoral payment mechanisms to ensure long-term sustainability, the study by Remers et al^1^ makes an important contribution. The study contributes not only to the evaluation of a specific intervention model but also to the conceptual advancement of integrated care strategies for people with dementia.

## Methodological Considerations and Critique

 Remers et al^1^ proceed with great methodological care. They clearly explain the central assumptions of their empirical models and acknowledge potential sources of bias. However, selection bias due to unobserved heterogeneity cannot be completely ruled out, because program participation was voluntary and regionally concentrated. This makes systematic differences between the intervention and control groups fundamentally possible. For example, differences may exist in the commitment of participating general practitioners or in the structural quality of the respective care regions. Remers et al^1^ are aware of this issue and conduct a detailed analysis of group differences prior to the intervention. However, selective participation may have introduced selection bias, potentially leading to an overestimation of the intervention’s effects.

 To further strengthen causal inference, I believe the complementary use of a difference-in-differences approach with pre-trend analysis would have been advisable. Even more, if researchers could rely on a longer time series, an event study approach including a graphical presentation of the outcomes over time (eg, for utilization indicators and costs) might be promising to assess dynamic program effects and to even enhance internal validity (See Miller^11^).

## Limitations and External Validity

 A key strength of the study is the use of detailed observational claims data from curative and long-term care, which allows a realistic description of healthcare utilization and costs. However, observational data are also associated with different restrictions that limit internal and external validity of the findings as noted previously.

 For instance, claims data contain less clinical and medical details than primary data from clinical studies. Thus, with observational data, it is challenging to assess dementia severity and the type of dementia accurately. Additionally, important factors such as cognitive abilities, the extent of functional impairment or informal support structures in the home environment are not directly captured in the available data. The consequences are twofold. First, it is not always possible to precisely identify dementia cases. Second, it might not be possible to control for important factors that might be relevant for the outcomes. Therefore, any causal estimation of the program impact must rely on strong identifying assumptions.

 One further potential limitation stems from the regional distribution of DementiaNet participants. This makes it necessary to adequately control for regional confounders. The results could be biased due to differences in local hospital capacities, service availability, or reimbursement levels—eg, as a result of regional price negotiations between health insurers and service providers.^12^ As noted by Remers et al,^1^ there is also the challenge of generalizing the findings to other healthcare systems where the gatekeeping role of general practitioners is less pronounced than in the Netherlands. Moreover, since the DementiaNet programme is currently mostly concentrated in the east and south of the Netherlands, where population ageing is relatively more advanced, the external validity of the findings may be limited in younger regions with different demographic and care needs.

 This study provides important foundations for future research. Qualitative studies could clarify how decision-making processes occur within network structures such as DementiaNet. Additionally, surveys could collect subjective information, such as satisfaction among caregivers, patients, and family members. Future research could also explore how DementiaNet could be expanded at the national level or transferred to other similarly organized healthcare systems (such as Germany). In addition, the potential effects of the DementiaNet programme could be explored in even greater detail using experimental or quasi-experimental designs. In particular, deeper insights into the programme’s underlying mechanisms would be informative. Indicators reflecting aspects of the decision-making process, identifying information flows or evaluating the quality of coordination could be particularly insightful. However, a significant challenge will be developing suitable empirical indicators that can reliably and accurately capture these complex processes and aspects. Finally, future research could also focus on possible spillover effects of care models such as DementiaNet on informal carers. This would shed more light on the welfare effects and thus the broader societal value of such integrated care models.

 In summary, despite its limitations, this study makes an important contribution to the literature by systematically evaluating DementiaNet, a potentially promising care model for a highly relevant social issue.

## Acknowledgements

 I would like to thank Luisa Licker and Xu Peng for their valuable comments.

## Ethical issues

 Not applicable.

## Conflicts of interest

 Author declares that he has no conflicts of interest.
